# Genome-wide identification of copy number variation using high-density single-nucleotide polymorphism array in Japanese Black cattle

**DOI:** 10.1186/s12863-016-0335-z

**Published:** 2016-01-25

**Authors:** Shinji Sasaki, Toshio Watanabe, Shota Nishimura, Yoshikazu Sugimoto

**Affiliations:** National Livestock Breeding Center, Odakura, Nishigo, Fukushima 961-8511 Japan; Shirakawa Institute of Animal Genetics, Japan Livestock Technology Association, Odakura, Nishigo, Fukushima 961-8061 Japan

**Keywords:** Copy number variation (CNV), BovineHD BeadChip Array, Japanese Black cattle

## Abstract

**Background:**

Copy number variation (CNV) is an important source of genetic variability associated with phenotypic variation and disease susceptibility. Comprehensive genome-wide CNV maps provide valuable information for genetic and functional studies. To identify CNV in Japanese Black cattle, we performed a genome-wide autosomal screen using genomic data from 1,481 animals analyzed with the Illumina Bovine High-Density (HD) BeadChip Array (735,293 single-nucleotide polymorphisms (SNPs) with an average marker interval of 3.4 kb on the autosomes).

**Results:**

We identified a total of 861 CNV regions (CNVRs) across all autosomes, which covered 43.65 Mb of the UMD3.1 genome assembly and corresponded to 1.74 % of the 29 bovine autosomes. Overall, 35 % of the CNVRs were present at a frequency of > 1 % in 1,481 animals. The estimated lengths of CNVRs ranged from 1.1 kb to 1.4 Mb, with an average of 50.7 kb. The average number of CNVR events per animal was 35. Comparisons with previously reported cattle CNV showed that 72 % of the CNVR calls detected in this study were within or overlapped with known CNVRs. Experimentally, three CNVRs were validated using quantitative PCR, and one CNVR was validated using PCR with flanking primers for the deleted region. Out of the 861 CNVRs, 390 contained 717 Ensembl-annotated genes significantly enriched for stimulus response, cellular defense response, and immune response in the Gene Ontology (GO) database. To associate genes contained in CNVRs with phenotypes, we converted 560 bovine Ensembl gene IDs to their 438 orthologous associated mouse gene IDs, and 195 of these mouse orthologous genes were categorized into 1,627 phenotypes in the Mouse Genome Informatics (MGI) database.

**Conclusions:**

We identified 861 CNVRs in 1,481 Japanese Black cattle using the Illumina BovineHD BeadChip Array. The genes contained in CNVRs were characterized using GO analysis and the mouse orthologous genes were characterized using the MGI database. The comprehensive genome-wide CNVRs map will facilitate identification of genetic variation and disease-susceptibility alleles in Japanese Black cattle.

**Electronic supplementary material:**

The online version of this article (doi:10.1186/s12863-016-0335-z) contains supplementary material, which is available to authorized users.

## Background

Copy number variation (CNV) is defined as deletions or duplications of genome segments ranging from 1 kb to several Mb [1]. Several studies have identified CNVs in cattle using three platforms: single-nucleotide polymorphism (SNP) arrays [2–9], comparative genomic hybridization (CGH) [10, 11], and next-generation sequencing (NGS) [12–15] (Additional file 1: Table S1). Previous results show that CNVs comprise a large fraction of the bovine genome, ranging from 3.29 to 498 Mb [2, 3, 5–15] (Additional file 1: Table S1).

CNVs can influence phenotypic variation or result in disease via mechanisms such as gene dosage modification and gene structure disturbance, either directly by exposing recessive alleles or indirectly by disturbing the regulatory regions of genes (for review, see [16]). Stranger et al. estimated that CNVs were responsible for 17.7 % of genetic variation in gene expression of human lymphoblastoid cell lines [17], and two studies on rodents showed that CNVs result in genome-wide expression changes in various tissues [18, 19], suggesting that CNVs alter gene dosage and are associated with phenotypic variance and disease susceptibility. In fact, several studies in cattle have demonstrated that CNVs contribute to phenotypic diversity in coat color [20] and milk production [21, 22], and also to diseases such as female fertility failure [21], nephritis [23], anhidrotic ectodermal dysplasia [24], myopathy [25], and osteopetrosis [26]. In addition, CNVs have been shown to contribute to phenotypic diversity and disease susceptibility in other species (for review, see [16, 27]).

Recently, substantial genotyping data using SNP arrays have been produced from genome-wide association studies [28] and genomic selection [29] in cattle, which can be directly exploited for CNV analysis. In particular, the Illumina Bovine High-Density (HD) BeadChip Array, with a total of 777,692 SNPs and an average marker interval of 3.4 kb, is 15-fold denser than the Illumina Bovine50K BeadChip Array [30], and provides higher resolution and a convenient screen for high-throughput CNV detection in the cattle genome [5, 8, 9].

Japanese Black cattle are highly rated owing to the abundant marbling of meat caused by intramuscular fat deposition [31]. Strict selection for marbling under a closed breeding system in Japan [32] has made the Japanese Black cattle genetically distinct from other cattle breeds [33]. A genome-wide map of CNVs has not been developed for Japanese Black cattle. Thus, to identify genotypic variability and disease-susceptibility alleles in the population, genome-wide CNV screens must be applied to this breed.

Therefore, this study aimed to investigate CNVs in Japanese Black cattle by performing a genome-wide screen of autosomes using genomic data from 1,481 animals analyzed with the Illumina BovineHD BeadChip Array.

## Results and discussion

### Genome-wide autosomal detection of CNVRs in 1,481 Japanese Black cattle

To identify CNVs in Japanese Black cattle, we used the Illumina BovineHD BeadChip Array, which contains 735,293 SNPs on autosomes with an average marker interval of 3.416 kb [30]. SNPs on sex chromosomes (X and Y) as well as on unknown chromosomes were excluded because of the lack of accurate information on their position in the bovine genome. In this study, a potential CNV was determined if it contained three or more consecutive SNPs [3, 5, 6, 9, 34] (Additional file 1: Table S1). To date, most SNP array-based CNV studies in cattle (Additional file 1: Table S1) and other animals (pig [35], chicken [36], sheep [37], and dog [38]) have used the hidden Markov model (HMM) approach-based PennCNV software [39, 40]. To identify CNVs and to compare our results with previous findings (Additional file 1: Table S1), we chose to use PennCNV software as well. We detected 55,593 CNV calls in 1,481 Japanese Black cattle. Of these, 1,099 singleton CNVs were identified, and 861 CNV regions (CNVRs) with overlapping CNVs [41] were detected in at least two animals. Since singleton CNVs were only detected in one animal, they were considered false positives compared to CNVRs [41] and excluded from subsequent analyses.

**Fig. 1:**
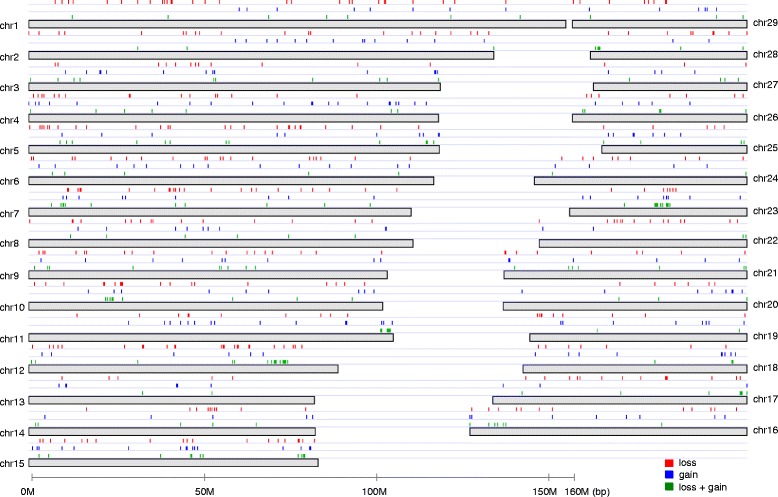
Distribution and status of CNVRs in the bovine genome. CNVRs (861 events, 43.65 Mb) in 1,481 Japanese Black cattle genotyped with the Illumina BovineHD BeadChip Array are shown on the autosomes in red (*loss*), blue (*gain*), and green (loss + gain). CNVR positions on the x-axis are based on the UMD3.1 assembly of the bovine genome

A total of 861 identified CNVRs covered 43.65 Mb of the UMD3.1 genome assembly, corresponded to 1.74 % of the 29 bovine autosomes, and consisted of 404 loss, 257 gain, and 200 loss plus gain (loss and gain within the same CNVR) events (Fig. 1, Additional file 1: Table S2). Overall, 35 % of the CNVRs were present at a frequency of >1 % in 1,481 animals (Additional file 1: Table S2). CNVs at a frequency of >1 % were characterized as copy number polymorphisms (CNPs) with potential involvement in the genetic basis of common phenotypes and diseases [1].

**Fig. 2:**
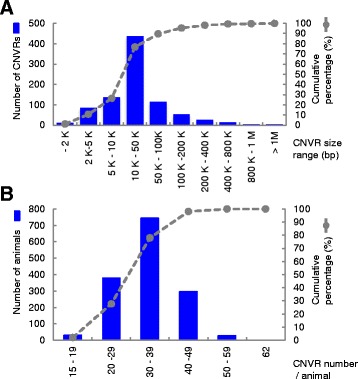
CNVR size distribution and CNVR number per animals. **a** The bar (*blue*) represents the CNVR size distribution in each size range; the line plot (*gray*) represents the cumulative percentage of CNVR number. **b** The bar (*blue*) represents the CNVR number per animal; the line plot (*gray*) represents the cumulative percentage of CNVR number

The estimated lengths of CNVRs ranged from 1.1 kb to 1.4 Mb with an average of 50.7 kb (Fig. 2a). Approximately 83 % of CNVRs were shorter than the average marker interval of the Illumina Bovine50K BeadChip Array in autosomes (68.3 kb) (Fig. 2a), indicating that a large number of small CNVRs were not detected in our population using the Illumina Bovine50K BeadChip Array. CNVRs were detected with an average of 35 events per animal (Fig. 2b). Detailed information of each CNVR is presented in Additional file 1: Table S2. In addition, we present the characteristics of CNVRs compared to previous studies in Additional file 1: Table S1.

### Comparison of current results with other cattle CNV studies

To characterize these CNVRs in more detail, we compared current results with three previously published cattle autosomal CNVRs using Illumina BovineHD BeadChip Arrays [5, 8, 9]. In addition, for a more comprehensive comparison, we also used the Database of Genomic Variants archive (DGVa) [42], including cattle CNV datasets from Illumina Bovine50K BeadChip Array [3, 4], CGH [11], and NGS [14], which were mapped on the UMD3.1 assembly of the bovine genome using dbVar [43]. The comparisons revealed that 72 % of the CNVR calls (620 CVNRs) detected in this study were within or overlapped with all combined non-redundant datasets (Table 2, detailed information for each comparison is presented in Additional file 1: Tables S2, S3, and S4). Comparisons with three previously reported CNV studies using the Illumina BovineHD BeadChip Array showed that CNVRs detected in this study overlapped 65.7 % of the CNVR calls reported by Hou et al. [5], 13.4 % of the CNVR calls reported by Jiang et al. [8], and 10.5 % of the CNVR calls reported by Zhang et al. [9] (Table 2 and Additional file 1: Table S2). The results were likely due to the different criteria used for the determination of CNV and the number of samples and breeds used in each study. CNV was defined as containing three or more consecutive SNPs in Hou et al. [5], in Zhang et al. [9], and in this study, whereas it was defined to contain 10 or more consecutive SNPs in Jiang et al. [8] (Additional file 1: Table S1). In the initial CNV discovery, we used three SNP windows, which is a less strict criterion than that in the study by Jiang et al. [8] (Additional file 1: Table S1). Therefore, we also inferred CNV with 5, 10, and 15 consecutive SNP windows, respectively (Additional file 1: Tables S1 and S2, Additional file 2). Of 861 CNVRs, 581 (67 %), 287 (33 %), and 181 (21 %) were also detected with 5, 10, and 15 consecutive SNP windows (Additional file 1: Table S2; Additional file 2), respectively. The average length of CNVRs in 3, 5, 10, and 15 consecutive SNP windows were 50.7, 65, 104, and 132 kb, respectively, indicating that there was an inverse correlation between the detection number of CNVRs and CNVR length, depending upon the size of SNP windows (Additional file 2). The increased number of consecutive SNPs may infer reliable CNV detection compared to a smaller number of SNPs, although the increased number of consecutive SNPs cannot capture small CNVRs. Thus, the results from this stepwise analysis of SNP windows will provide valuable information for further analyses, including experimental validation of CNVRs. Detailed SNP window information for each CNVR is presented in Additional file 1: Table S2.

In addition to CNV definition, the inconsistency noted among studies could also be due to differences in sample size and cattle breeds. Our study used 1,481 animals of a single cattle breed, while Hou et al. [5] used 674 animals of 27 different cattle breeds, and Jiang et al. [8] and Zhang et al. [9] used 96 and 6 animals of a single cattle breed (Additional file 1: Table S1), respectively. The present study used a larger number of animals than the studies by Jiang et al. [8] and Zhang et al. [9] from a single breed; therefore, the current CNV screen may be more effective than previous intrabreed studies [8, 9].

In this study, we did not calculate the proportion of unique CNVRs in Japanese Black cattle directly; however, 28 % of identified CNVRs have not been previously reported in non-redundant CNV datasets (Table 1) and might be unique in Japanese Black cattle. As reported in dogs [44, 45] and horses [46], integration of current CNV data with the results from different cattle breeds will facilitate the identification of unique genotypic variability and disease-susceptibility alleles in Japanese Black cattle.

**Table 1:** Comparison of 861 CNVRs detected in this study with results from three other CNV studies used the Illumina BovineHD BeadChip Array and structural variants deposited in the DGVa

		Overlapped CNVR of this study	
data	studies	Count	Percentage of count
SNP-based Studies	Hou et al. [5]	566	65.7 %
BovineHD BaseChip^a^	Jiang et al. [8]	115	13.4 %
	Zhang et al. [9]	90	10.5 %
DGVa^b^		280	32.5 %
All combined non-redundant datasets^c^		620	72 %

^a^BovineHD BeadChip Array contains 735,293 SNPs on autosomes

^b^DGVa, the Database of Genomic Variation Archive [42]

^c^Datasets derived from Hou et al. [5], Jiang et al. [8], Zhang et al. [9], and DGVa

### Experimental validation of CNVRs by quantitative PCR and CNVR_27 by PCR with flanking primers

**Fig. 3:**
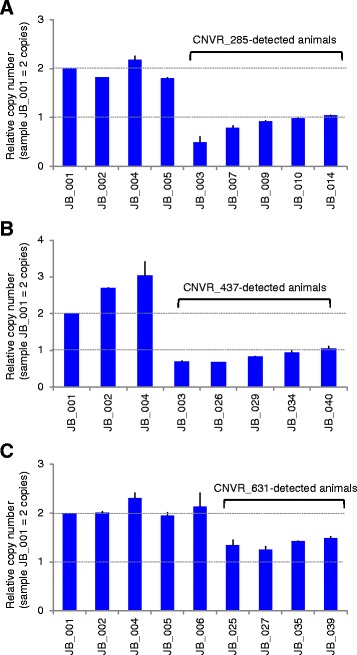
Quantitative PCR (qPCR) validation of CNVR_285 (**a**), CNVR_437 (**b**), and CNVR_631 (**c**). The left-most bar in each panel represents a calibrator animal (JB_001). The calibrator animal is assumed to contain two copies of the DNA segment detected from the PennCNV analysis. The Basic transcription factor 3 gene (*BTF3*), which served as an internal qPCR standard for both copies at a locus (2n), was co-amplified with the primers. The x-axis represents the animals. The brackets represent CNVR-detected animals using the Illumina BovineHD BeadChip Array. Error bars represent ± Standard Error of Mean (SEM) obtained from triplicate experiments

Quantitative PCR (qPCR) was performed to verify CNVR calls using the SNP array as an independent experimental validation. Three loss-type CNVRs, CNVR_285, CNVR_437, and CNVR_631, were selected. The Basic transcription factor 3 gene (*BTF3*), which served as an internal qPCR standard for both copies at a locus (2n) [2], was co-amplified with the primers. The copy number estimated by qPCR was approximately one (Fig. 3), which was in agreement with the expected copy number estimated by the PennCNV analysis using the Illumina BovineHD BeadChip Array platform.

**Fig. 4:**
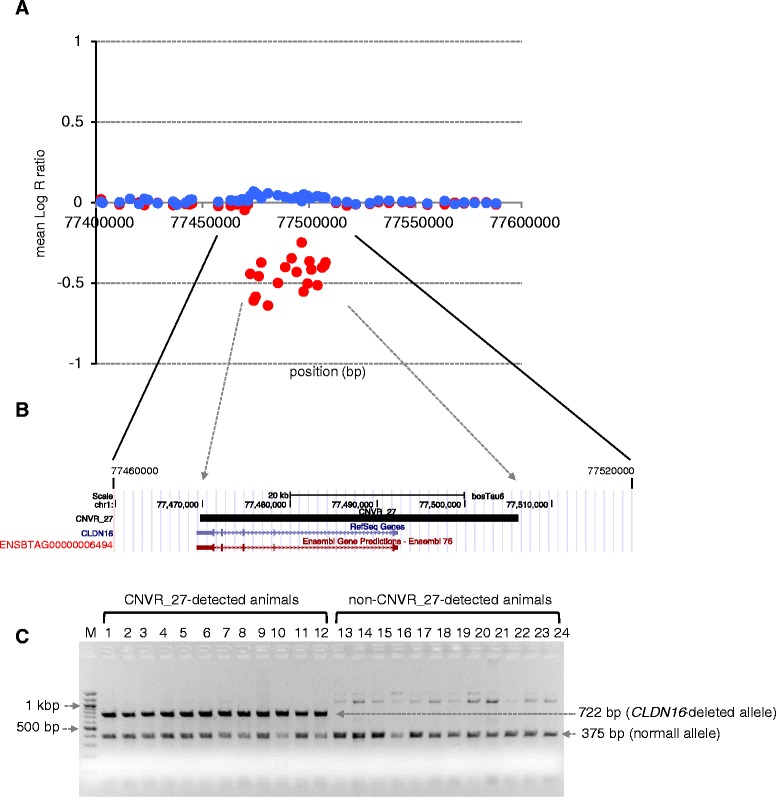
CNVR_27 overlapping with *CLDN16* gene region on chromosome 1. **a** Regional SNP plot of CNVR_27. The mean log R ratio of CNVR_27 animals (*red*) and the mean log R ratio of non-CNVR_27 animals (*blue*). The mean log R ratio was calculated from 116 animals. SNP positions were based on the UMD3.1 assembly of the bovine genome. **b** CNVR_27 was visualized using the UCSC Genome Browser [62]. The RefSeq gene symbol and Ensembl IDs of *CLDN16* were labeled. **c** Detection of *CLDN16*-deletion allele in CNVR_27-detected animals using PCR with flanking primers for the deleted region. CNVR_27-detected animals (lane 1 to 12) and non-CNVR_27-detected animals (lane 13 to 24), which were inferred using PennCNV analysis with the Illumina BovineHD BeadChip Array. The PCR product size of the *CLDN16*-deletion allele is 722 bp and that of the wild type allele is 375 bp. M, 100 bp ladder marker

To further test the CNV calls derived from the PennCNV analysis with Illumina BovineHD BeadChip Array, we examined a region of Claudin 16 gene (*CLDN16*) on chromosome 1. Hirano et al. reported that the first four exons of *CLDN16* were deleted in Japanese Black cattle with autosomal recessive nephritis [23]. We found that CNVR_27 overlapped with *CLDN16* (Fig. 4a, b, Additional file 1: Tables S2, S5). The mean Log R ratio of 22 SNPs, which were consecutively located within a 36,382 bp window between *BovineHD0100022316* (77,469,795 bp) and *BovineHD0100022336* (77,506,177 bp) on chromosome 1, was decreased in 116 animals (Fig. 4a). To further confirm whether animals with CNVR_27 have the *CLDN16* deletion, we performed PCR with flanking primers designed to amplify the *CLDN16* deletion region in CNVR_27-detected animals. A *CLDN16-*deletion allele was detected in CNVR_27-detected animals, whereas this deletion was not detected in non-CNVR_27-detected animals (Fig. 4c). These results also indicated that the PennCNV analysis with Illumina BovineHD BeadChip Array inferred reliable CNVs in this study.

### Gene content of CNVRs

Of 861 CNVRs, 390 were within or overlapped with 717 Ensembl genes, including 651 protein-coding genes, 17 small nuclear RNAs, 5 small nucleolar RNAs, 1 ribosomal RNA, 15 microRNAs, 23 unprocessed pseudogenes, 3 processed pseudogenes (a processed pseudogene is a pseudogene that lacks introns and is thought to arise from reverse transcription of mRNA followed by reinsertion of DNA into the genome [47]), and two miscellaneous RNAs (Additional file 1: Tables S2 and S5). Out of 390 CNVRs, 334 contained exons, and the remaining 56 were intronic CNVRs (Additional file 1: Table S2). Of 334 CNVRs, 125 contained exons derived from multiple protein-coding genes, ranging from 2 to 21 Ensembl genes, suggesting that these CNVRs influence the function of multiple genes.

Although the 717 Ensembl-annotated genes had a wide spectrum of molecular functions (Additional file 1: Table S5), gene ontology (GO) analysis using the PANTHER classification system [48, 49] showed that the most over-represented biological processes were stimulus response, cellular defense response, and immune response (Table 2 and Additional file 1: Table S6). Of these, 63 olfactory receptors function as smell sensors (Additional file 1: Table S6), and have been frequently reported CNVs in cattle (for review, see [16]). In addition, the immune-related gene enrichment within CNVs was in agreement with previously reported bovine CNV studies (for review, see [16]). In particular, 6 CNVRs were clustered within a 3.17 Mb window from 25,325,230 bp to 28,494,393 bp on BTA23 (Fig. 1), including the major histocompatibility complex (MHC) gene family members (Additional file 1: Table S6). In addition, 12 CNVRs were clustered within a 2.97 Mb window from 22,208,187 bp to 25,173,443 bp on BTA10 (Fig. 1), including T-cell receptors (Additional file 1: Table S6), which are generated by gene rearrangement in somatic cells.

**Table 2:** Gene Ontology (GO) categories significantly overrepresented in CNVRs

	GO categories^a^	gene number in CNVR	expected gene number^b^	*P* value^c^
Biological	response to stimulus	124	61.18	8.14E-12
Process	cellular defense response	51	15.71	1.25E-10
	immune system process	120	61.91	4.61E-10
	hemopoiesis	23	5.57	5.45E-06
	immune response	51	22.36	1.83E-05
	B cell mediated immunity	28	8.8	3.71E-05
	extracellular transport	18	4.94	1.01E-03
	response to interferon-gamma	14	3.1	1.14E-03
Cellular	immunoglobulin complex	20	0.98	4.32E-18
Component	protein complex	48	17.26	2.21E-08
	macromolecular complex	50	21.76	4.03E-06
	heterotrimeric G-protein complex	8	1.24	2.23E-03
	MHC protein complex	10	2.03	2.53E-03
Molecular	antigen binding	20	0.98	1.35E-17
Function	ATPase activity, coupled to transmembrane movement of substances	9	1.46	3.40E-03

^a^List of GO categories associated with overrepresentation of genes in the CNVRs (*P* < 0.05)

^b^Ensembl gene list of 19,799 Bos taurus genes compared to the list of 717 genes in the CNVRs using PANTHER 9.0

^c^Bonferroni-corrected *P* value

### Phenotypic information of mouse orthologous genes in CNVRs

The use of mouse models has proven to be one of the most powerful approaches to understand *in vivo* gene functions [47, 50]. The Mammalian Phenotype (MP) Ontology in Mouse Genome Informatics (MGI) is the most comprehensive phenotypic database that enables the annotation of phenotypes in a genetic context [51–53]. Thus, in addition to GO term analysis, to associate bovine genes contained in CNVRs with phenotypes, we converted 560 bovine Ensembl gene IDs to their 438 orthologous associated mouse gene IDs (MGI IDs) (Additional file 1: Table S7) using BioMart in both Ensembl and MGI [53, 54]. Several different bovine Ensembl gene IDs were connected with a single MGI ID, such as T cell receptor family and olfactory receptor family (Additional file 1: Table S7), and several bovine Ensembl gene IDs did not connect with a MGI ID (Additional file 1: Table S7); therefore, the number of converted mouse orthologous genes was reduced. Out of 438 MGI IDs, 195 were assigned to 1,627 phenotypic categories in MP IDs (Additional file 1: Table S8). This list will provide useful information for understanding phenotypic implications of CNV events *in vivo*. In particular, knockout mice for bovine orthologous genes in loss-type CNVRs may provide important information on phenotypic expression of CNV events in cattle. For instance, CNVR_27 is a loss-type CNVR exons of *CLDN16* (Fig. 4, Additional file 1: Tables S2 and S5), and the mouse orthologous gene (MGI:2148742) was assigned to 9 phenotypic categories in MP IDs (Additional file 1: Table S8), such as abnormal renal reabsorbtion and abnormal renal calcium reabsorbtion. These symptoms are consistent with those of null deletions of *CLDN16* in cattle [55, 56].

## Conclusions

In this study, we identified 861 CNVRs in 1,481 Japanese Black cattle using the Illumina BovineHD BeadChip Array. Of these, 72 % of CNVR calls were within or overlapped with previously reported cattle CNVs. Experimentally, three CNVRs were validated using quantitative PCR, and one CNVR was validated using PCR with flanking primers specific to the deleted region. These results suggest that the current analysis inferred reliable CNV calls. Out of 861 CNVRs, 390 contained 717 Ensembl-annotated genes, which are significantly enriched for stimulus response, cellular defense response, and immune response in the Gene Ontology (GO). In addition to the GO analysis, we characterized the mouse orthologous genes using the MGI database to associate bovine genes contained in CNVRs with phenotypes. This list will provide useful information for understanding their implication in the CNV events *in vivo*. The comprehensive genome-wide CNVRs map generated by this study will facilitate the identification of genotypic variability and disease-susceptibility allelles in Japanese Black cattle.

## Methods

### Ethics statement

All animal experiments were performed according to the Guidelines for Care and Use of Laboratory Animals of Shirakawa Institute of Animal Genetics, and this research project was approved by the Shirakawa Institute of Animal Genetics Committee on Animal Research (H21-2). We have obtained the written agreement from the cattle owners to use samples and data.

### Sample collection and genotyping

Genomic DNA from Japanese black cattle was isolated from blood samples of 787 cows, adipose tissue samples of 591 steers, and semen samples of 103 bulls.

Genomic DNA of all samples was genotyped using the Illumina BovineHD BeadChip Array (Illumina, cat#WG-450-1002), which contains 735,293 autosomal SNPs [30] (for SNP intervals, see Additional file 3), according to the manufacturer’s instructions. SNP clustering and genotype calling were performed using GenomeStudio version 2011 (Illumina, version 1.9.4), and all markers passed quality control (call rate > 98 %). The UMD3.1 assembly was used to map SNP positions [57].

### Identification of CNVs

The ratio of observed normalized intensity of the experimental sample to the expected intensity of each locus (log R ratio: LRR) and the allelic intensity ratio (B allele frequency: BAF) of samples were reported using GenomeStudio. The population frequency of B allele (PFB) was generated based on the BAF of each SNP in the population. To identify CNVs, we used PennCNV software (version June 2011) [39, 40], which incorporates factors including LRR, BAF, marker distance, and PFB into a hidden Markov model.

In this study, only autosomes were used for the detection of CNVs. Genomic waves were adjusted for the GC content of the 1 Mb genomic region (500 kb each side) surrounding each SNP. Samples with a standard deviation of logR ratio > 0.3, BAF drift > 0.01, and wave factor > 0.05 were excluded from the analysis. We also removed CNV calls with a confidence score < 10, calls in the 15-kb centromeric and telomeric regions (Additional file 1: Table S9) [58], and calls in the immunogloblin region [59] (Additional file 1: Table S10). T-cell receptor family members were not excluded from the analysis due to mapping uncertainty. In this study, a potential CNV was determined if it contained three or more consecutive SNPs [3, 5, 6, 9, 34]. The union region of overlapping CNVs detected in at least two animals was defined as a CNV region (CNVR) [41].

### Comparison of current results with other cattle CNV studies

We compared CNVRs detected in this study with results from three other CNV studies that used the Illumina BovineHD BeadChip Array [5, 8, 9]. We also compared CNVRs with structural variants deposited in the DGVa [57] and dbVar [43] databases.

### Quantitative PCR validation of CNVR

Real-time qPCR was performed for CNVR validation using the 7900HT Real-Time PCR system (Applied Biosystems). Primers and probes were designed for three CNVRs (Additional file 1: Table S11). Amplification reactions (20 μl⋅well^-1^) were carried out in triplicate with 20 ng of genomic DNA, 1× Absolute QPCR ROX Mix (Thermo Scientific, cat#AB-1138/B), 400 nM of each primer, and 200 nM of each probe. The *BTF3*, which served as an internal qPCR standard for both copies at a locus (2n) [2], was co-amplified with the primers (Additional file 1: Table S12). Three replicate reactions were performed for each primer pair, and a comparative C_T_ method was used to calculate the copy number [2]. ∆ C_T_ was calculated by subtracting the *BTF3* C_T_ value from the sample C_T_ value for each replicate. The average ∆ C_T_ value for the three replicates was calculated. To determine the ∆∆ C_T_, the average ∆ C_T_ of a calibrator animal, which had two copies of the DNA segment, was used. Finally, the copy number was given using the formula 2 × 2 ^-∆∆ CT^.

### PCR validation of Claudin 16 (*CLDN16*) deletion

PCR was performed as described by Hirano et al. [23]. The following forward and reverse primer pairs were used: DN-F (5′-TATGCTGTTGATGTTTATGTAG-3′)/DN-R (5′-CCCCCCCCCGCCTTTTTC-3′) to detect the wild type allele, and DA-F (5′-ATTGTATTTTTAGGAGTGACTC-3′)/DA-R (5′-CCCCCCCCCACTCTATAC-3′) to detect the *CLDN16* deletion allele. 

### Gene annotation and Gene Ontology (GO) analysis

Gene content of CNVRs was assessed based on the gene annotation of the UMD3.1 genome assembly using Ensembl (Cow release 77) [54]. The PANTHER classification system (PANTHER 9.0) [48, 49] was used to assess the probability of overrepresented genes in CNVRs within biological process, cellular composition, and molecular function using Bonferroni correction for multiple comparisons.

### Phenotype annotation

To obtain phenotypic information of genes in the CNVRs, the mouse orthologs of bovine genes in the CNVRs were obtained from the Mouse Genome Information (MGI) resource [53]. Before the analysis, bovine Ensembl gene IDs were converted to their mouse orthologous Ensembl gene IDs or MGI IDs using BioMart in both Ensembl and MGI [53, 54]. Phenotypic annotations in MGI were obtained from MGI_PhenoGenoMP.rpt.txt [60] and VOC_MammalianPhenotype.rpt.txt [61].

## Availability of supporting data

The datasets supporting the results of this article are included within the article and its additional files.

## Additional files

Additional file 1: Table S1. Characterization of cattle autosomal CNVRs based on previously published studies. **Table S2** – Detailed features of 861 autosomal CNVRs identified in this study. **Table S3** – Comparison of CNVRs detected in this study with the results from three other CNV studies using the Illumina BovineHD BeadChip Array. **Table S4** – Comparison of CNVRs detected in this study with structural variants deposited in the DGVa. **Table S5** – Detailed features of Ensembl genes in CNVRs. **Table S6** – PANTHER pathway analysis of genes in CNVRs in this study. **Table S7** – The corresponding MGI gene to Bovine Ensembl gene in CNVRs. **Table S8** – Detailed phenotypic description of mouse orthologous genes for Bovine Ensembl genes in CNVRs. **Table S9** – Detailed features of excluded centromeric and telemeric regions in this study. **Table S10** – Detailed features of excluded immunoglobulin regions in this study. **Table S11** – Primers and probes for CNVR validation. **Table S12** – Primers and probes for the *basic transcriptional factor 3*. (XLSX 686 kb) Additional file 2:
**Detection number and mean size of CNVRs in 3, 5, 10, and 15 consecutive SNP windows.** (PPTX 53 kb) Additional file 3:
**Autosomal SNPs intervals of Illumina BovineHD BeadChip Array.** (PPTX 48 kb)

## Abbreviations

CNV: copy number variation
CNVR: CNV region
PCR: polymerase chain reaction
SNP: single nucleotide polymorphism
