# Supplementary material for: Genome-wide identification of copy number variation using high-density single-nucleotide polymorphism array in Japanese Black cattle
Source: BMC Genet. 2016 Jan 25;17:26. doi: 10.1186/s12863-016-0335-z (PMC4727303; doi:10.1186/s12863-016-0335-z)
Supplement: Additional file 2: — Detection number and mean size of CNVRs in 3, 5, 10, and 15 consecutive SNP windows. (PPTX 53 kb) [file 12863_2016_335_MOESM2_ESM.pptx]

## Slide 1
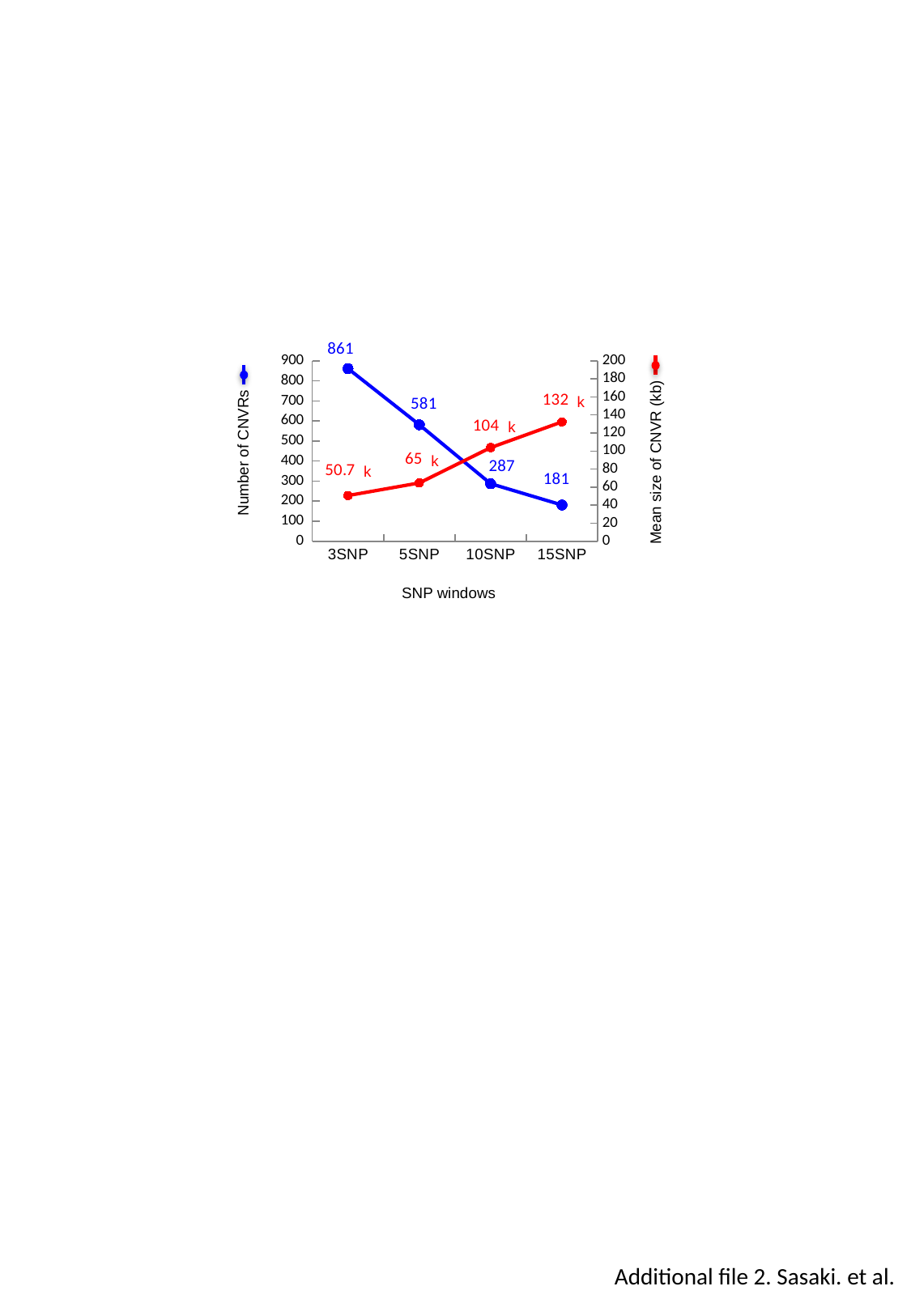

### Chart
| Category | number of CNVRs | 累積比率％ |
|---|---|---|
| 3SNP | 861.0 | 50.7 |
| 5SNP | 581.0 | 64.66 |
| 10SNP | 287.0 | 103.826 |
| 15SNP | 181.0 | 132.357 |
k
k
Number of CNVRs
Mean size of CNVR (kb)
k
k
SNP windows
Additional file 2. Sasaki. et al.
