# Supplementary figures and images for: Genome-wide identification of copy number variation using high-density single-nucleotide polymorphism array in Japanese Black cattle
Source: BMC Genet. 2016 Jan 25;17:26. doi: 10.1186/s12863-016-0335-z (PMC4727303; doi:10.1186/s12863-016-0335-z)

## Slide 1
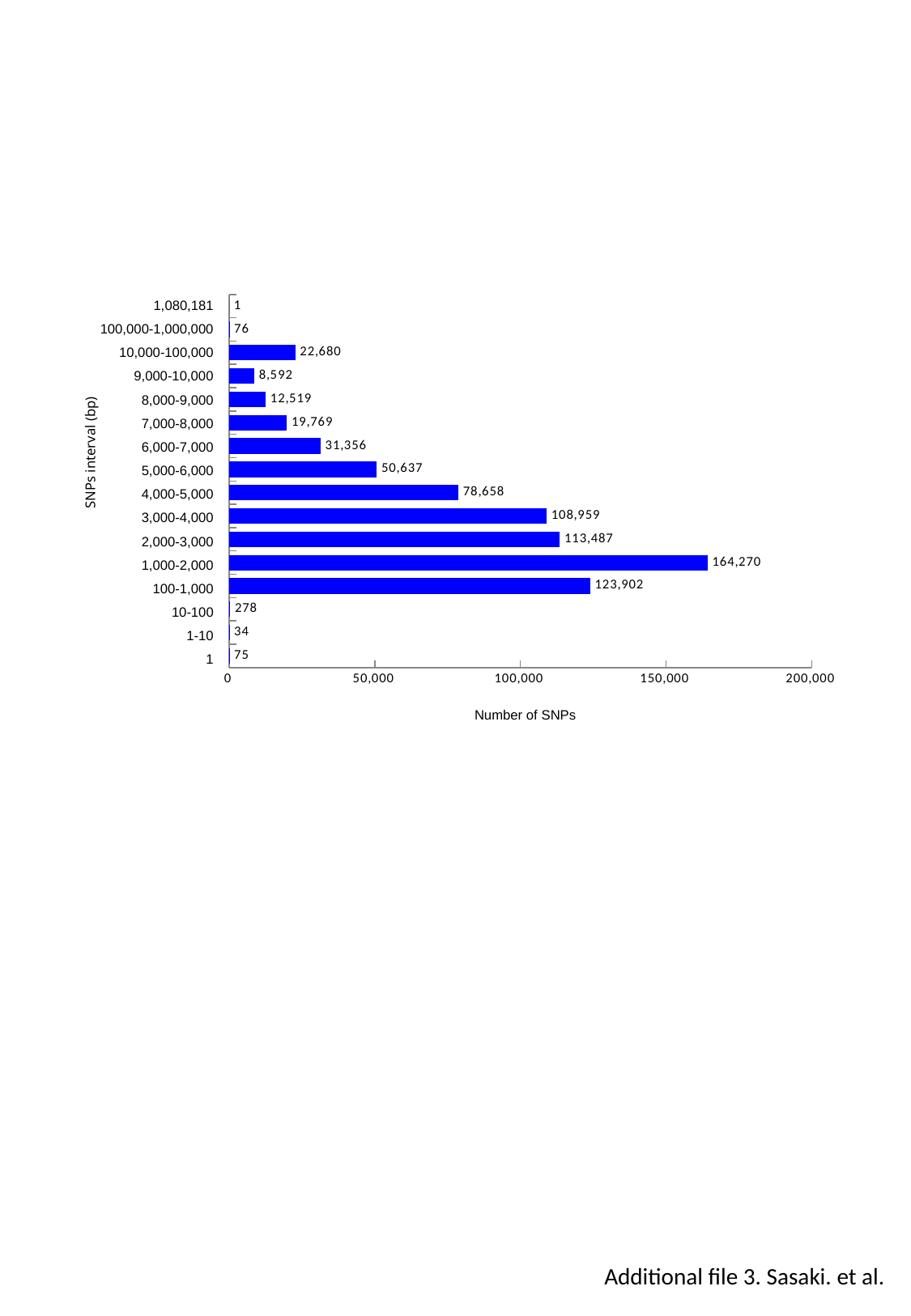

1,080,181
100,000-1,000,000
10,000-100,000
9,000-10,000
8,000-9,000
7,000-8,000
6,000-7,000
5,000-6,000
4,000-5,000
3,000-4,000
2,000-3,000
1,000-2,000
100-1,000
10-100
1-10
1
### Chart
| Category | 度数 |
|---|---|
| 1.0 | 75.0 |
| 10.0 | 34.0 |
| 100.0 | 278.0 |
| 1000.0 | 123902.0 |
| 2000.0 | 164270.0 |
| 3000.0 | 113487.0 |
| 4000.0 | 108959.0 |
| 5000.0 | 78658.0 |
| 6000.0 | 50637.0 |
| 7000.0 | 31356.0 |
| 8000.0 | 19769.0 |
| 9000.0 | 12519.0 |
| 10000.0 | 8592.0 |
| 100000.0 | 22680.0 |
| 1.0E6 | 76.0 |
| 1.080181E6 | 1.0 |SNPs interval (bp)
Number of SNPs
Additional file 3. Sasaki. et al.

Supplement: Additional file 3: — Autosomal SNPs intervals of Illumina BovineHD BeadChip Array. (PPTX 48 kb) [file 12863_2016_335_MOESM3_ESM.pptx]
